# Diseases of the Skin

**Published:** 1903-06

**Authors:** 


### BOOK REVIEWS.

Diseases of the Skin. Their Description, Pathology, Diagnosis and Treatment. With special reference to the Skin Eruptions of Children and an Analysis of 15,000 cases of Skin Disease. By H. Radcliffe Crocker, M. D. (Lond.), F. R. C. P., Physician for Diseases of the Skin in University College Hospital; Honorary Member of the American Dermatological Society; Examiner in Medicine, Apothecaries' Hall, London. Third edition, revised and enlarged. With 4 plates and 112 illustrations. Octavo, pp. 1,466. Philadelphia: P. Blakiston's Son & Co. 1903. [Price, $5.00.]

It is now ten years since the last edition of Crocker's Diseases of the Skin was issued. This period has been fruitful concerning the science of medicine in general and especially so as relates to dermatology. The necessity has therefore arisen of bringing forward what is new in reference to the subject. Besides, the last edition has been out of print for a number of years, and it was time that it should reappear with up-to-date additions. It is true that the work has been in some sense supplanted by American works, notably those of Hyde and Stelwagen; we, nevertheless, welcome the new edition on account of its former prestige and the certainty that, in its new form, it will prove of value to the profession. The new edition is large, containing 1,466 pages, about twice the number in the old edition, making the volume too heavy and somewhat unhandy, although this could hardly have been avoided considering this vast accumulation of new material. Still, the publishers might have prevented this objection by issuing the work in two volumes, as was done in the case of the foreign edition.

It would almost seem that the authors must have rewritten the entire book, for not alone have new subjects been exhaustively treated, but many admirable changes have been made in the description of old diseases, largely in the way of amplification, a necessity of growing out of the old order of things. In certain instances, there is a lack of conciseness and point, but the bulk of the work is perhaps sufficiently excellent to excuse this single blemish.

The work of revision must have been laborious, almost equal to the original compilation. References are quite numerous, so that the reader need not necessarily confine himself to the author's views. Of course, many of the subjects discussed are sure to be much more interesting to the specialist than to the student and general practitioner. For instance, under the title of lichen variegatus, the author has included the following affections: parakeratosis variegata (Unna), dermatitis variegata (Boeck), psoriasiform and lichnoid exanthum (Neisser and Jadasohn), erythrodermie pityriasique en plaques (Brocq), and pityriasis lichenoides chronica (Julensberg). There can be no doubt that these several conditions are closely allied one to another, but there are still many points concerning which questions hard to answer have arisen, although the able paper of Fox and Macleod has thrown much light upon the subject. They claim that the original term parakeratosis variegata is most fitly chosen, saying that the primary lesion is not always a papule. This view, of course, conflicts with Crocker's present definition, viz: lichen variegatus.

We notice that Crocker has dropped the name of hydroa herpetiformis conferred by Tilbury Fox, and has written the name of dermatitis herpetiformes over that interesting disease. He wrongfully gives to Fox the right of discovery, for the full honors in this instance are due to Duhring for his classic exposition.

Leukemia cutis is treated under the separate headings of leukemia cutis, chloroma and pseudoleukemia cutis. This seems unnecessary, as the changes of the blood are little understood and the pathology of the skin changes are so much alike that it would seem that these cognate conditions might all be grouped under one heading. In treating seborrhoic dermatitis, under the heading of seborrhoides, the author has followed French authority. While citing Unna's opinion and that of Sabourand, and giving their chief points, he, nevertheless declares that it must be left to the future to show which interpretation is correct.

The histology and pathology of the various diseases of the skin, which were so carefully considered in the former edition, have not alone been amplified, but all those relating to new cutaneous diseases are elaborately discussed—like blastomycosis, due to the blastomycites, which has been inoculated into animals and from the lesion fungi have been inoculated and cultivated. In regard to the streptococcic organ of impetigo contagiosa, the author, who was the first to describe the organism now proceeds more carefully. It is obvious, he argues, that further research is still necessary, and he suggests that only fluid from unruptured vesicles and media liquid be employed and considers that to continue to make observations on fluid from beneath crusts is so obviously open to error as to be unscientific and a waste of time. He takes up the different clinical forms and lays considerable stress on the impetigo of Bockhart, a variety which we have here lately recognised, as well as another variety, impetigo contagiosa gyrata, which apparently is rarely seen in England, although it is quite common in the United States. He says that the schizomyceties, which includes the bacteria and micrococci, are proving more and more to be the cause of inflammatory disease and granuloma. The psorosperms are no longer to be considered a pathogenic agent in the skin, having proved to be metamorphosed epithelial cells. In comparing the general drug treatment of the last edition with the present, but slight changes appear. The more marked of these apply to mechanical means—electricity clearly leading the march of improvement. The new modes of treatment are fully discussed, notably $x$-ray and the Finsen light. What Crocker says upon these subjects derives, of course, its chief value from the experiences had and the results obtained by others. He advises the use of high tubes with a spark gap of ½-foot while treating diseases of the skin.

A few new illustrations have been added to the previous small collection. The colored one on syphilis has no special value and interferes with the binding. The one on ringworm is much better. The weakest feature of the book consists in its illustrations. However, any criticism upon a work of this character seems almost an impertinence, seeing that it is well known to almost every practitioner as the heretofore standard work in the English language. At one time it had no rival; but in the absence of new editions, and the strong hold upon the profession taken, in the meantime, by the admirable works of Hyde and Stelwagen, the conclusion is forced that hereafter, as relates to these several authors, the honors must be easy.

